# Aberrant Right Subclavian Artery: A Case Report

**DOI:** 10.7759/cureus.107747

**Published:** 2026-04-26

**Authors:** Jessica Meechan, Elena Chavez, Jessica Lai, Camryn Long, Myles A Nelson, Edie L Sperling

**Affiliations:** 1 Medical Anatomical Sciences, Western University of Health Sciences, Lebanon, USA

**Keywords:** aberrant right subclavian artery (arsa), cadaver case report, cardiac anatomy/pathologic anatomy, clinical and functional anatomy, variant arterial anatomy

## Abstract

During routine dissection of a medical cadaver, an aberrant right subclavian artery (ARSA) was identified originating from the aortic arch. An ARSA, present in 0.2-2.5% of the population and often asymptomatic, increases the risk of surgical nerve injury during head, neck, and thoracic surgeries. ARSAs can also impinge on the esophagus and cause dysphagia or odynophagia, impinge on the trachea and cause dyspnea, or be impinged by the esophagus and/or trachea, limiting blood supply to the upper extremity. Most often, ARSAs follow a retroesophageal course, as in this case. In addition to the ARSA, the vertebral arteries were also variant and branched directly off the common carotid arteries, as opposed to the subclavian arteries, and the right recurrent laryngeal nerve was nonrecurrent. This case describes a donor with three anatomical anomalies to highlight the clinical relevance of recognizing ARSA and related variants and how to identify signs and symptoms of a symptomatic ARSA.

## Introduction

A typical right subclavian artery arises from the brachiocephalic trunk, which also gives rise to the right common carotid artery. An aberrant right subclavian artery (ARSA) is a rare anatomical variation with a pooled prevalence rate of 0.2% to 2.5%; left aberrant subclavian artery pathways also exist [1]. ARSAs may be more common in females than males, although the evidence is mixed [1,2]. ARSA can follow one of three paths: (1) pretracheal (4.2-5% of cases), (2) between the trachea and esophagus (12.7-15%), or (3) retroesophageal (80-84%) [3].

Embryologically, the proximal part of the right subclavian artery originates from the right fourth pharyngeal arch, and the distal part originates from the right dorsal aorta and the right seventh intersegmental artery [4]. If both the fourth arch and the right dorsal aorta involute, the right subclavian artery instead arises from the right seventh intersegmental artery and distal right dorsal aorta [4]. This occurs in the eighth week of gestation [4]. The formation of an ARSA often occurs in individuals with Down syndrome (35% of cases) or other genetic abnormalities (3% of cases) [4].

Depending on the course of an ARSA, it can contribute to a variety of symptoms, although most cases are asymptomatic [5]. If pretracheal, it has been found to cause airway issues, including dyspnea, chronic cough, and respiratory complications; if passing between the trachea and esophagus, it can compress the esophagus, causing dysphagia or odynophagia; in this position or retroesophageal, the ARSA itself can be compressed, causing decreased blood supply to the right upper extremity [2,6]. Symptoms of arterial compression have been noted as early fatigue with upper extremity activities [2,6]. Tracheal and esophageal symptoms are more common than upper extremity symptoms [2].

Surgery is performed if symptoms are concerning, emergent, and/or if a Kommerell diverticulum or thoracic aortic aneurysm is found to be present. Total, open, and hybrid endovascular approaches have all been performed with varied results. A Kommerell diverticulum is an aneurysm at the base of an aberrant subclavian artery and occurs in approximately 60% of individuals with the variant [2]. Individuals also present with aneurysms in the distal arch or proximal descending thoracic aorta. The presence of an aneurysm increases the risk for aortic dissection, embolism, rupture, and upper extremity ischemia. Aberrant subclavian arteries are most often identified due to symptoms (most commonly dysphagia) and subsequent investigation, although they can also be diagnosed asymptomatically with imaging for a separate issue [2]. Insertion of pacemaker leads is performed through the subclavian vein with real-time ultrasound or fluoroscopy; an aberrant subclavian artery may or may not be noted at that time.

Imaging of the head, neck, and chest reveals aberrant subclavian arteries and associated aneurysms and is typically the first step in diagnosing dyspnea, chronic cough, dysphagia, or odynophagia. Computed tomography (CT) is most common, but further vascular imaging can include magnetic resonance angiography (MRA), duplex ultrasound, echocardiography, and pulmonary function tests. Patients may also undergo barium swallow tests or upper endoscopy [2].

A study involving 49 patients who underwent surgery for a right or left aberrant subclavian artery reported the specific surgeries and success rates; 46 of the patients were symptomatic. An ARSA was present in 78% (n=38), and the remaining patients had an aberrant left subclavian artery (22%, n=11). Eighty-four percent (n=41) had a Kommerell diverticulum, and 22% (n=11) had a distal aortic arch or proximal descending thoracic aortic aneurysm, with some having both. The aberrant subclavian arteries were addressed with transposition in 65% of patients (n=32), a carotid to subclavian bypass in 22% of patients (n=11), and an ascending aorta to subclavian bypass in 12% of patients (n=6). The Kommerell diverticulum was addressed by resection in 39% of patients (n=19). Thirty-one percent (n=15) underwent replacement of the distal arch or proximal descending thoracic aorta [2].

While symptoms this donor may have experienced are unknown, the case of an ARSA with associated vertebral artery variants in someone without other apparent genetic variants is quite rare and presents an opportunity to contribute to the general knowledge on ARSA morphology for the surgical field. This article was previously presented as a meeting abstract at the Oregon Physicians & Surgeons of Oregon Annual Presentation on September 12, 2025.

## Case presentation

**Figure 1:**
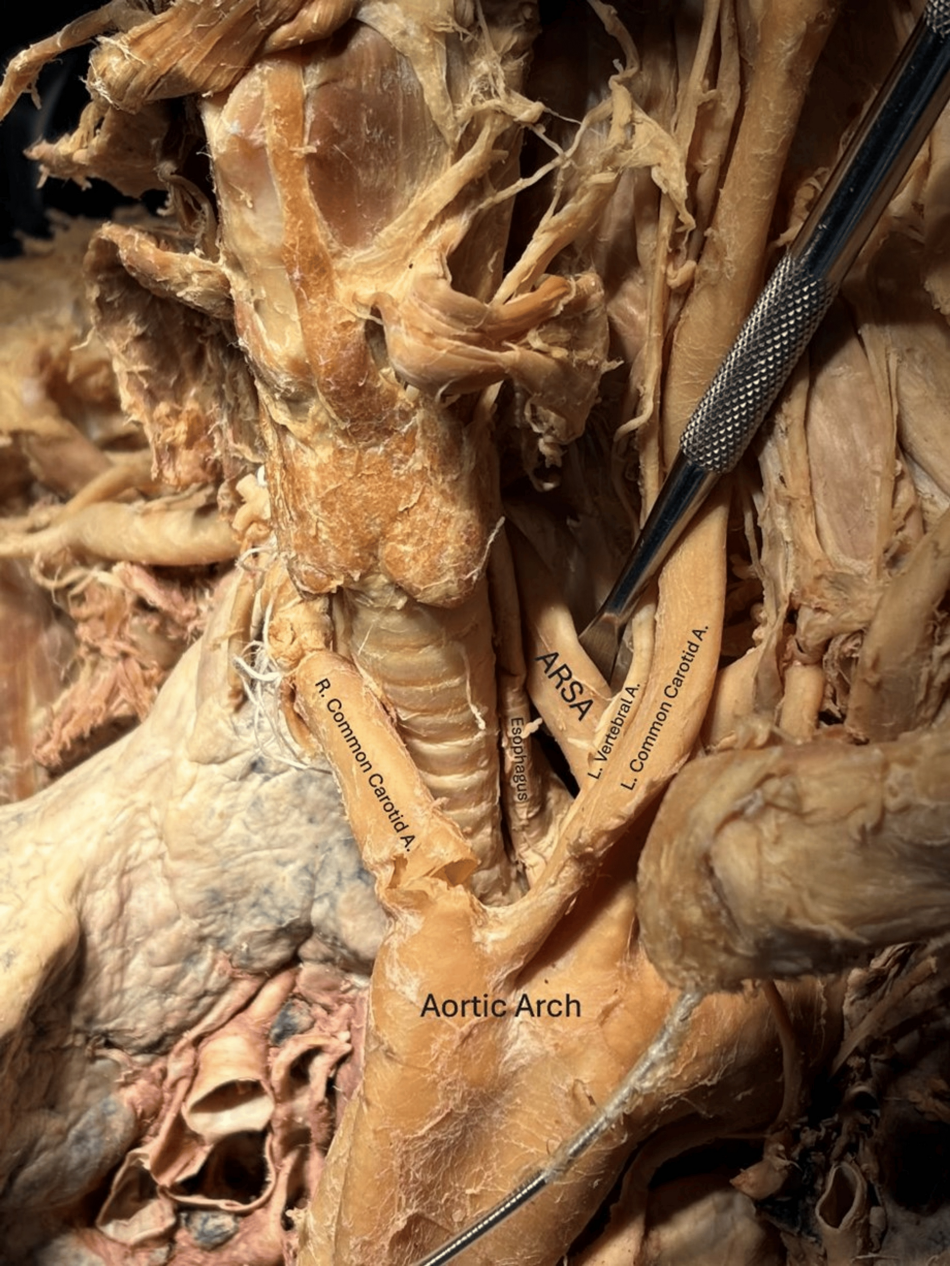
The aberrant right subclavian artery can be seen branching posterior to the left subclavian artery and traveling posterior to the esophagus; the left vertebral artery is seen branching from the posterior left common carotid artery. A: artery, ARSA: aberrant right subclavian artery, L: left, R: right.

**Figure 2:**
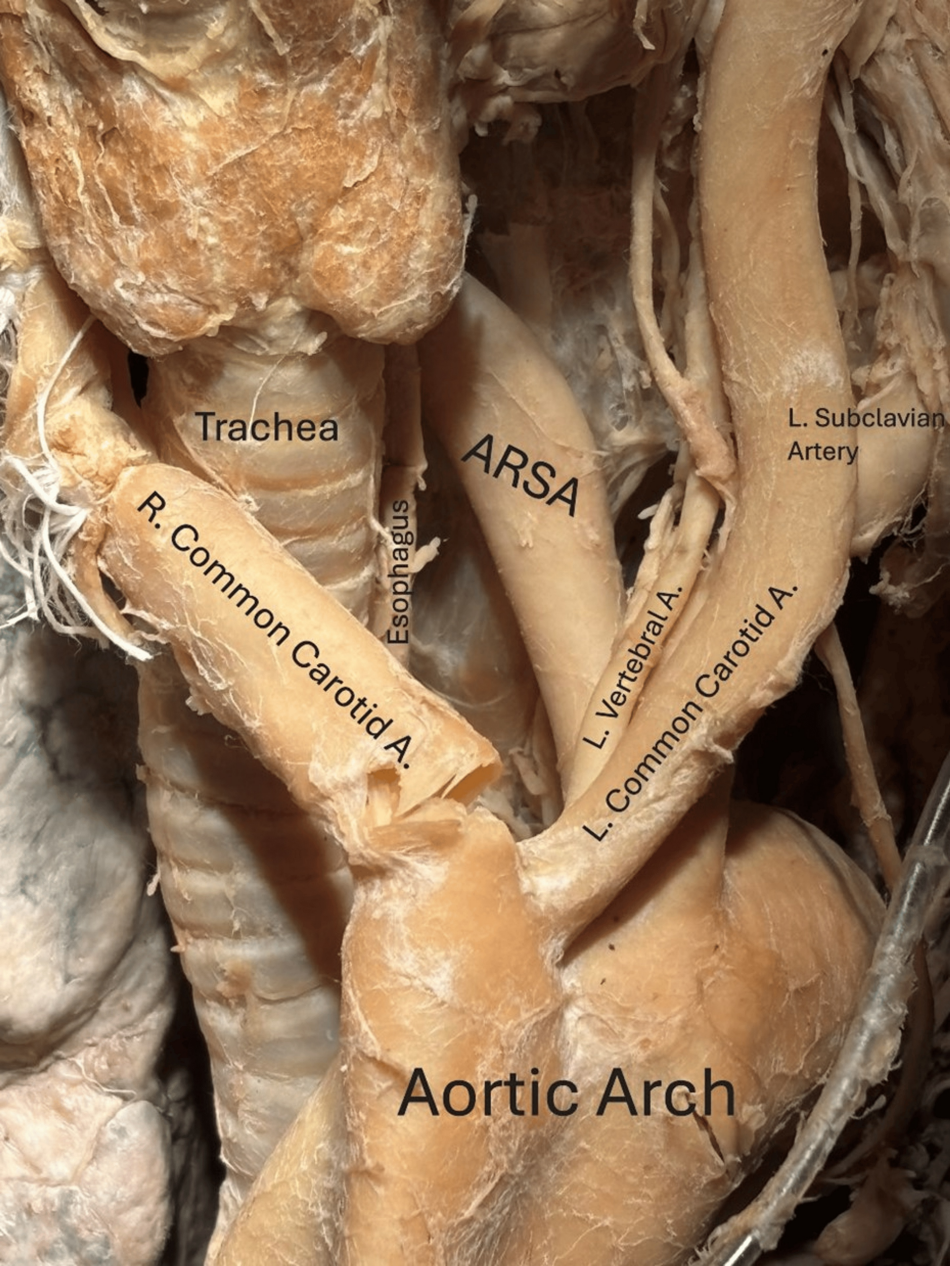
The aberrant right subclavian artery's retroesophageal route, beginning posterolaterally to the left subclavian artery and crossed anteriorly by the left vertebral artery and left common carotid artery. A: artery, ARSA: aberrant right subclavian artery, L: left, R: right.

**Figure 3:**
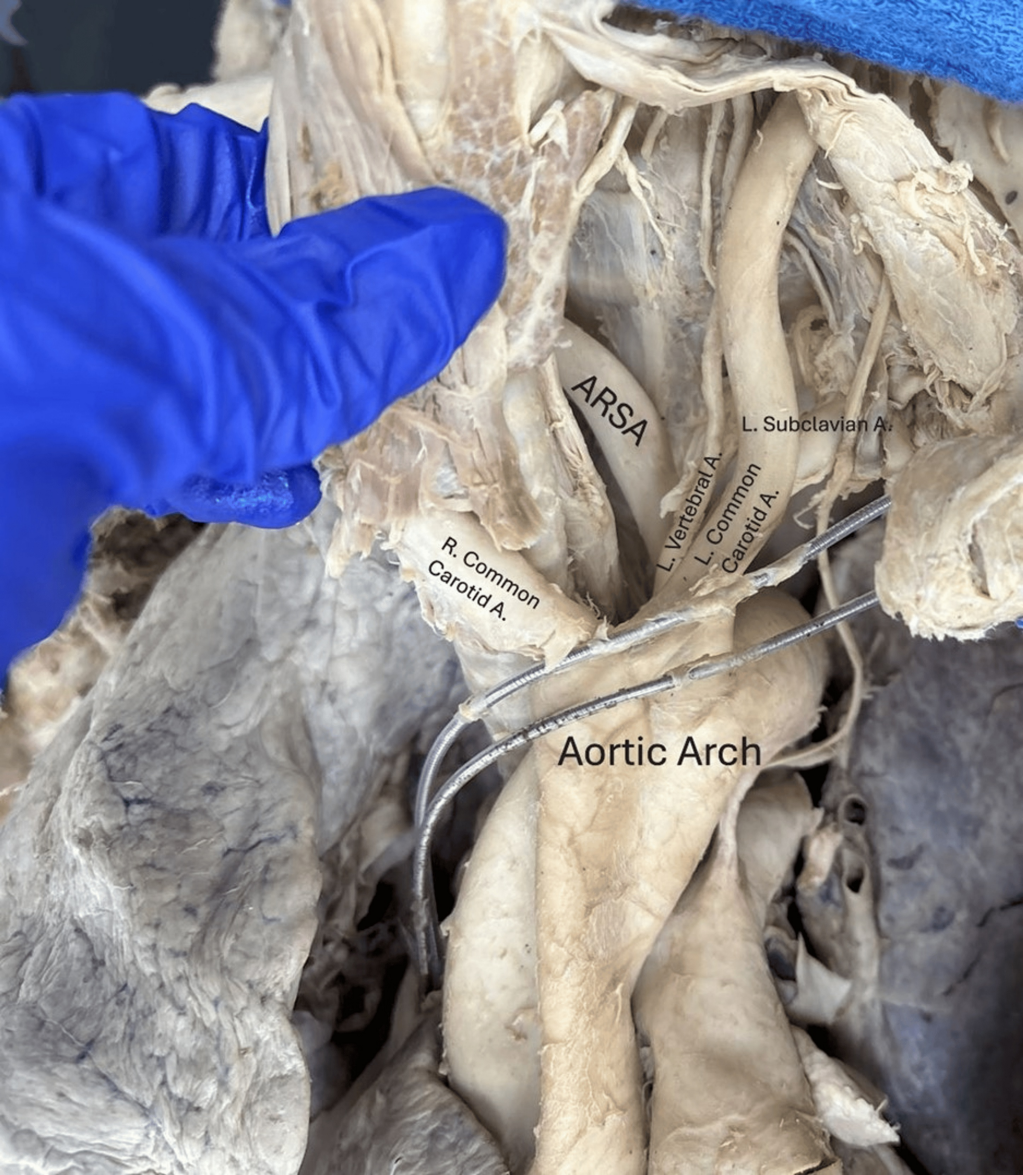
Branches off the aortic arch, from donor's right to left in direction of blood flow: right common carotid (from which branched the right vertebral artery, not visible), left vertebral artery branching from the left common carotid artery, left subclavian artery, and the aberrant right subclavian artery. Parts of a pacemaker can also be seen. A: artery, ARSA: aberrant right subclavian artery, L: left, R: right.

**Figure 4:**
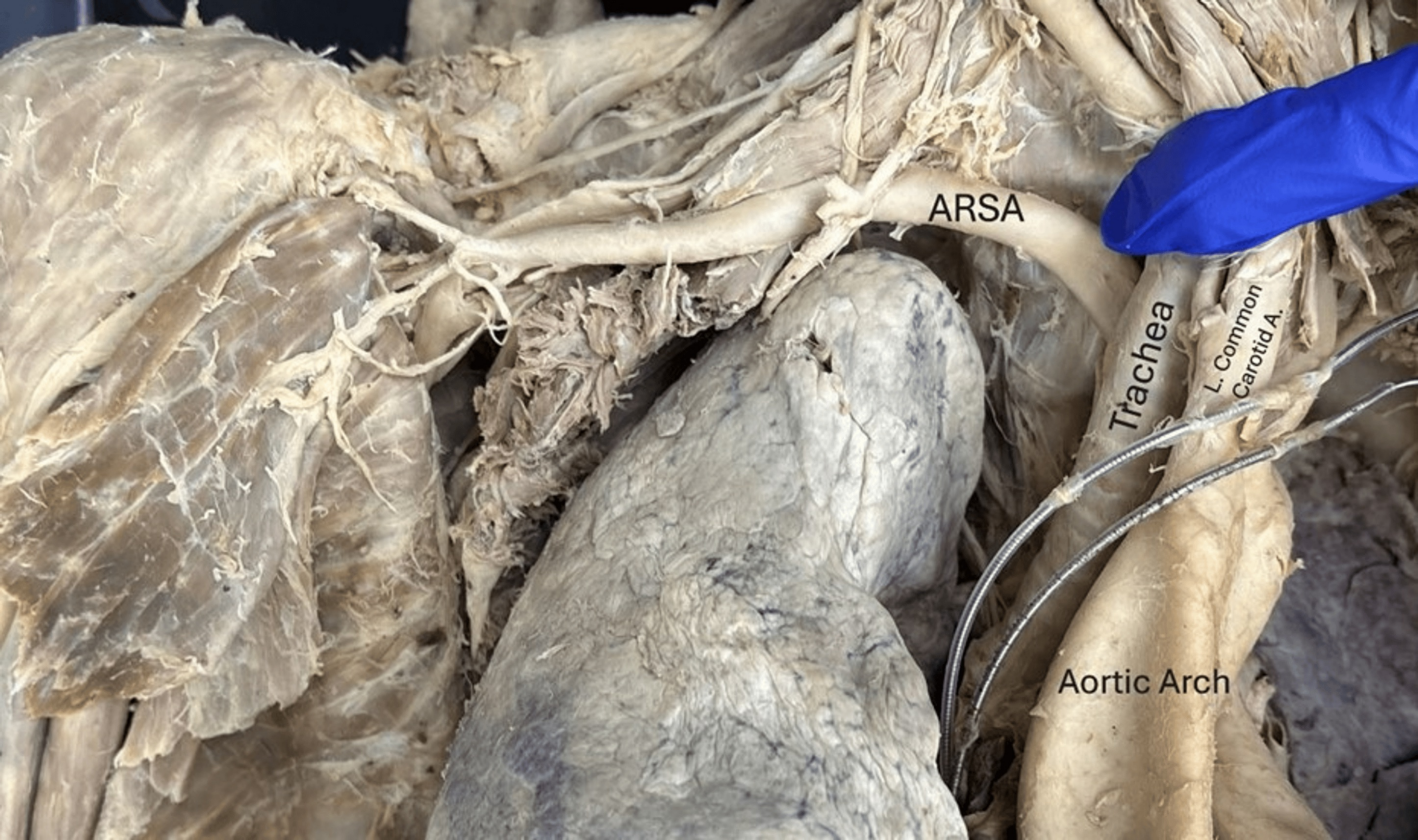
The aberrant right subclavian artery emerging from posterior to the trachea and esophagus, where it traveled in a typical pathway posterior to the clavicle (removed), anterior scalene (removed) and right subclavian vein (removed). Rope used during the embalming process to tie off the right common carotid artery is visible on the right subclavian artery. Parts of the pacemaker are visible. A: artery, ARSA: aberrant right subclavian artery, L: left, R: right.

**Figure 5:**
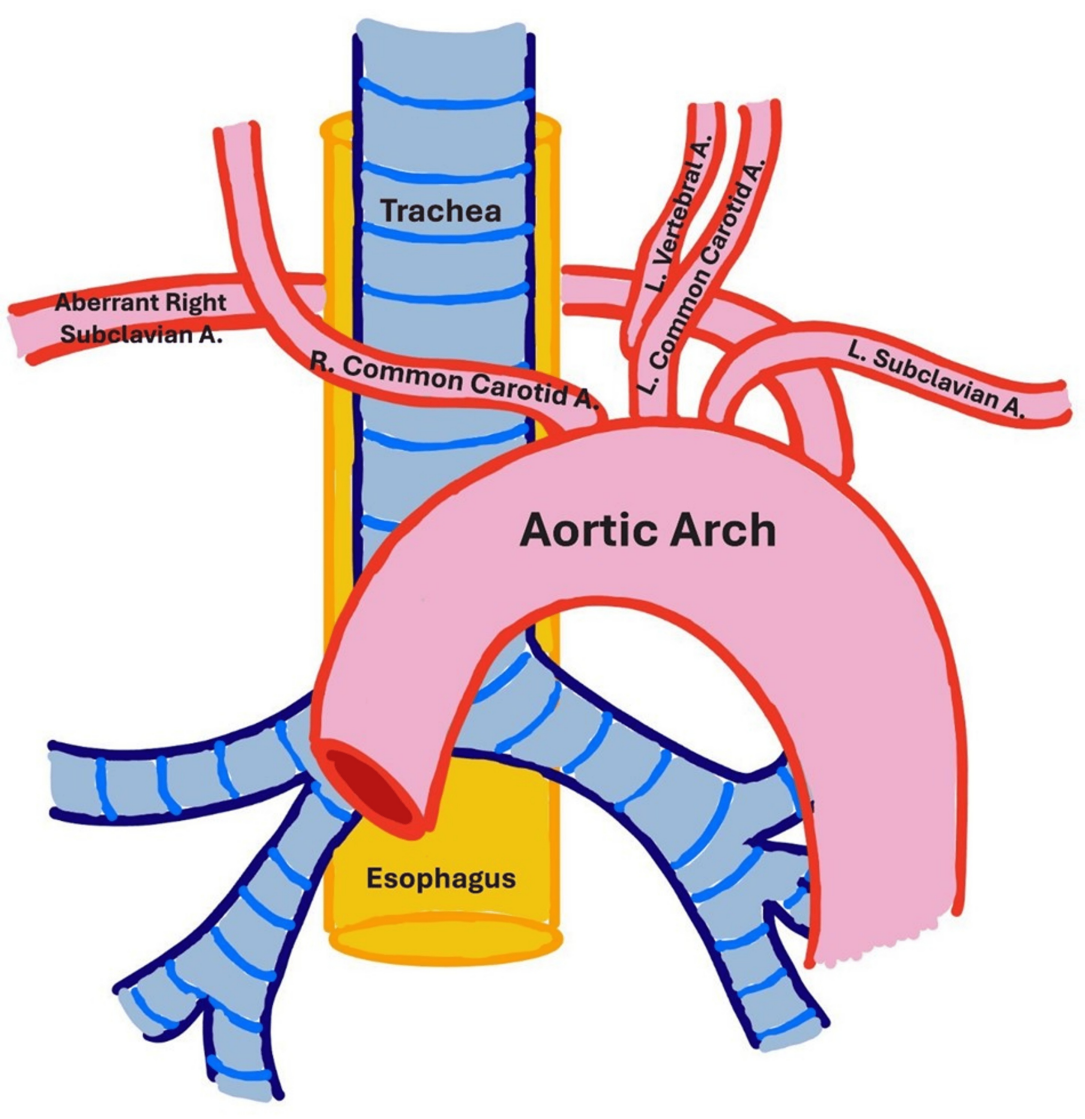
Schematic of the aberrant right subclavian artery and associated branching: there was no brachiocephalic trunk, so the right common carotid artery branched directly off of the aortic arch, the vertebral arteries branched off of the common carotids bilaterally instead of off of the subclavian arteries, and the aberrant right subclavian artery branched posterolaterally to the left subclavian artery instead of off of the brachiocephalic trunk. A: artery, L: left, R: right.

During routine medical cadaver dissection at the Western University of Health Sciences College of Osteopathic Medicine of the Pacific-Northwest, an ARSA was identified in an otherwise typical, 87-year-old female donor. The ARSA originated from the aortic arch, 4 mm lateral to the left common carotid artery and posterior to the left subclavian artery, with 2 mm overlap (Figures 1, 2). The ARSA arose at an 87-degree angle, almost directly superior. It traveled posterior to the trachea and esophagus. The vertebral arteries branched directly off the common carotid arteries, as opposed to the subclavian arteries, as is typical (Figures 1, 2). The vertebral arteries branched 4 mm superior to the base of the respective common carotid arteries (Figures 1, 2; right vertebral artery not visible). No Kommerell diverticulum or aortic arch or descending thoracic aortic aneurysms were observed; a pacemaker was present subcutaneously on the left upper thorax, with leads traveling in the left subclavian vein, left brachiocephalic vein, and into the right atrium and ventricle. The full branching pattern from the aortic arch can be seen in Figure 3. Once the ARSA crossed the midline, it was typical in its branching pattern into the upper extremity, posterior to the clavicle, right subclavian vein, and anterior scalene (Figure 4). A schematic of the ARSA and associated vasculature can be seen in Figure 5.

The right recurrent laryngeal nerve was found to be nonrecurrent, branching directly from the right vagus nerve into the posterior cricoarytenoid muscle. The thoracic duct was typical, shifting from right to left in the thorax, posterior to the esophagus, and entering the left venous angle. A pacemaker was situated subcutaneously on the upper left chest wall, with leads traveling through the left subclavian vein, left brachiocephalic vein, and into the right atrium and ventricle. Deep dissection to the retropharyngeal space was performed, with the deep neck flexors as the deepest structures visualized. See Table 1 for a summary of the variants in this case. 

**Table 1:** Description of typical anatomy and this case's variant anatomy for the three variants found in the donor: right subclavian artery, bilateral vertebral arteries, and left recurrent laryngeal nerve. R: right, L: left.

Structure	Typical Anatomy	Case’s Variant Anatomy
Right subclavian artery	Branches from the brachiocephalic trunk, which is the first branch of the aortic arch (R to L, direction of blood flow)	Fourth branch of the aortic arch after the R common carotid, L common carotid, and L subclavian artery (Figures 1-5)
Vertebral arteries	Branch from the subclavian arteries, posterolateral to the common carotid arteries bilaterally	Branched from the common carotid arteries bilaterally, approx. 4mm superior to the base of the common carotids (Figures 1-5)
R recurrent laryngeal nerve	Branches from the R vagal nerve in the thorax on the anterolateral R subclavian artery to travel underneath and then posterior to the artery, traveling superiorly in the R tracheoesophageal groove in the neck and into the posterior cricoarytenoid muscle on the posterior larynx	Branched from the right vagal nerve in the neck at the level of the larynx and enters directly into the posterior cricoarytenoid muscle (not visualized in figures)

## Discussion

The thickness (caliber) of typical subclavian arteries is approximately 6.0 ± 1.1 mm [7], and because subclavian arteries are so close to the heart, arterial pressure is high [8]. The esophagus is an easily compressible muscular tube, closed except when a bolus passes through during swallowing, whereas the trachea is held open by cartilaginous rings. However, the cartilaginous rings are C-shaped and open on the posterior aspect to allow for tracheal flexibility and temporary compression by a bolus in the esophagus, so the large, high-pressure subclavian artery passing posterior to the trachea or esophagus can cause impingement on the fibroelastic membrane and trachealis muscle [9]. Theoretically, a retrotracheal ARSA (between the esophagus and trachea) would cause compression of both the esophagus and trachea, a retroesophageal ARSA would cause compression primarily of the esophagus, and a pretracheal ARSA might be asymptomatic against the cartilaginous rings [3]. Similarly, arterial compression would theoretically be highest in a retroesophageal ARSA, which is pressed against the anterior vertebral bodies, and less so in the pretracheal ARSA, which could expand anteriorly [3]. In this retroesophageal case, it may be surmised that, if symptomatic, esophageal and upper extremity symptoms may have been most likely to present.

Nonrecurrent right recurrent laryngeal nerves are commonly found with ARSAs [10]. Typical recurrent laryngeal nerves loop around the right subclavian artery before traveling in the tracheoesophageal groove toward the posterior larynx [10]. When an ARSA is formed, the embryological process of the recurrent portion of the recurrent laryngeal nerve does not occur, leaving it as a simple branch off of the vagus nerve, as in this case [10]. Its altered path is an important consideration in surgical thyroid or esophageal interventions; presurgical imaging (CT, MRI, MRA, ultrasound) would identify an aberrant subclavian artery and raise the index of suspicion for a variant recurrent laryngeal nerve.

Subclavian artery stenosis may be another factor to consider when treating individuals with ARSAs. Subclavian artery stenosis, usually due to atherosclerosis, is a form of peripheral artery disease (PAD), which is common in older adults (~2% prevalence in subclavian arteries in the adult population). Subclavian artery stenosis is significant because of its higher morbidity, contributing to ischemia of the upper extremities, brain, and heart. Typically, left subclavian artery stenosis is more common due to the left subclavian artery’s origin directly off the aorta, but an ARSA that also arises directly off the aorta increases the risk of stenosis on the right side [11].

Less investigation has been conducted into the thoracic duct implications of ARSAs. Thoracic duct anomalies often occur alongside ARSAs but may be missed intraoperatively; damage to the thoracic duct during surgery can result in chylothorax, a serious condition [10]. Thoracic duct variants alongside ARSAs have been documented, including right-draining thoracic ducts and duplicate thoracic ducts [12]. Assessing the thoracic duct position prior to surgery is necessary to avoid injury. A typical thoracic duct was present in this case, shifting from right to left in the mid-thorax posterior to the esophagus and entering the left venous angle.

Important limitations of this case include limited clinical information (n=1), no health history, and a lack of imaging.

## Conclusions

This case study presents a single cadaveric donor with three anatomical variants with clinical implications: an ARSA arising posterolateral to the left subclavian artery from the aortic arch, bilateral vertebral arteries arising from the respective common carotid arteries, and a right nonrecurrent laryngeal nerve. No health history or imaging was available, limiting interpretation. These anatomical variants can impact surgical interventions in the neck, shoulder, and thorax, including thyroidectomies, esophageal surgery, and cardiac surgery. While not present in this case, aortic aneurysms and thoracic duct anomalies are commonly found with ARSAs. Although there are several pathways that an ARSA can take, this ARSA followed a retroesophageal course. The presence of an ARSA may be associated with clinical manifestations such as dysphagia, dyspnea, stridor, chest pain, or cough. However, the majority of ARSA cases remain asymptomatic, making diagnosis in living patients challenging. To avoid surgical complications, awareness of this anatomical variation is of high importance when considering surgical approaches involving nearby structures, including the esophagus, thyroid, and thoracic aorta.

## References

[1] Polednak AP (2017). Prevalence of the aberrant right subclavian artery reported in a published systematic review of cadaveric studies: the impact of an outlier. Clin Anat.

[2] Tallarita T, Rogers RT, Bower TC, Stone W, Farres H, Money SR, Colglazier JJ (2023). Characterization and surgical management of aberrant subclavian arteries. J Vasc Surg.

[3] Natsis K, Didagelos M, Gkiouliava A, Lazaridis N, Vyzas V, Piagkou M (2017). The aberrant right subclavian artery: cadaveric study and literature review. Surg Radiol Anat.

[4] Buffoli B, Verzeletti V, Hirtler L, Rezzani R, Rodella LF (2021). Retroesophageal right subclavian artery associated with a bicarotid trunk and an ectopic origin of vertebral arteries. Surg Radiol Anat.

[5] Brauner E, Lapidot M, Kremer R, Best LA, Kluger Y (2011). Aberrant right subclavian artery- suggested mechanism for esophageal foreign body impaction: case report. World J Emerg Surg.

[6] Polguj M, Chrzanowski Ł, Kasprzak JD, Stefańczyk L, Topol M, Majos A (2014). The aberrant right subclavian artery (arteria lusoria): the morphological and clinical aspects of one of the most important variations--a systematic study of 141 reports. ScientificWorldJournal.

[7] Arnett DM, Lee JC, Harms MA (2018). Caliber and fitness of the axillary artery as a conduit for large-bore cardiovascular procedures. Catheter Cardiovasc Interv.

[8] Bazaral MG, Nacht A, Petre J, Lytle B, Badhwar K, Estafanous FG (1988). Radial artery pressures compared with subclavian artery pressure during coronary artery surgery. Cleve Clin J Med.

[9] Downey RP, Samra NS (2026). Anatomy, thorax, tracheobronchial tree. StatPearls [Internet].

[10] Nusrath S, Basudhe M, Raju K (2024). Non-recurrent laryngeal nerve and variant thoracic duct anatomy in the presence of an aberrant right subclavian artery during esophageal cancer surgery. Indian J Surg Oncol.

[11] Caesar-Peterson S, Bishop MA (2026). Subclavian artery stenosis. https://www.ncbi.nlm.nih.gov/sites/books/NBK470221/.

[12] Oya S, Mine S, Watanabe M, Yamada K (2015). Oesophageal cancer with an aberrant right subclavian artery accompanied by a thoracic duct anomaly. Eur J Cardiothorac Surg.

